# Chidamide-based 3-drug combination regimen reverses molecular relapse post transplantation in AML1-ETO–positive acute myeloid leukemia

**DOI:** 10.3389/fphar.2022.1059930

**Published:** 2023-01-13

**Authors:** Yang Xi, Li Chenglong, Zhang Rong, Wang Wen, Wang Yu, Chen Jiao, Huang Juan, Che Feifei, Xiao Rong, Jiang Tao, Li Hui, Huang Xiaobing

**Affiliations:** ^1^ Sichuan Provincial People’s Hospital, Affliated Hospital of University of Electronic Science and Technology of China, Chengdu, China; ^2^ Sichuan Provincial People’s Hospital (Medical Group), Dongli Hospital, Chengdu, China

**Keywords:** chidamide, 3-drug combination, AML1-ETO, post-transplantation, relapse

## Abstract

**Objective:** We aimed to explore a new method to reverse early relapse in patients with AML1-ETO–positive acute myeloid cell transplantation.

**Methods**: A chidamide-based 3-drug combination regimen was used in our center to treat patients with AML1-ETO–positive AML post transplantation but negative flow cytometry results. A retrospective analysis was performed of the survival rate and possible influencing factors of patients with relapse treated with this regimen in our center from January 2018 to January 2022.

**Results**: The overall response rate was 95.8% (23/24), and the median number of treatment courses was 4 (range, 3–12 courses). The total molecular complete response (MCR) was 79.1% (19/24) after all treatments, and the molecular complete response was 37.5% (9/24) after one cycle of treatment but reached 58.3% (14/24) after four cycles; overall, the proportion of MCR increased gradually with the increase in treatment cycles. The projected 5-year overall survival rate was 73.9%. The projected 5-year leukemia-free survival rate was 64.8%, and the projected 1-year cumulative relapse rate was 35.5%. The incidence of grade II–IV graft-versus-host diseases (GVHD) was 29.2% (7/24), and that of grade III–IV GVHD was 20.8% (5/24), which could be effectively controlled by glucocorticoid therapy combined with calcineurin inhibitors The total incidence of chronic GVHD was 29.2% (7/24), and all cases were localized chronic GVHD. The total infection rate was 33.3% (8/24), mainly involving bacterial and fungal infections, and the incidence of life-threatening infections was 4.17% (1/24). The treatment-related mortality rate was 0%; and the total mortality rate was 20.8% (5/24). Nausea and vomiting, thrombocytopenia, and neutropenia were common adverse reactions, all of which were Common Terminology Criteria for Adverse Events grade 2–3 events and reversible after drug withdrawal. In terms of immunity, Th1 cell counts gradually increased, Th17 cell counts gradually decreased, and the Th1/Th17 ratio gradually increased after treatment. The CD8^+^ T lymphocyte count increased gradually, while the CD4^+^ T lymphocyte count did not change significantly.

**Conclusion:** Our chidamide-based 3-drug combination regimen led to a high remission rate and tolerable adverse reactions in patients with AML1-ETO–positive post-transplant relapse, and most patients can achieve long-term survival with this regimen.

## 1 Introduction

AML1-ETO is determined by t (8; 21) (q22; Q22) chromosomal translocation of tumor proteins, which are seen in 1%–5% of patients with acute myeloid leukemia, most often in younger patients. According to ELN stratification, t (8; 21) (q22; Q22) cases may be classified into the good prognosis group. Studies in China have shown that the HAA regimen can increase the rate of early remission in induction therapy among AML1-ETO–positive AML patients, but about 40% of patients relapse within 2 years (Zhu et al., 2016). A number of real-world studies in China have confirmed that patients with continuous positive AML1-ETO have a poor prognosis and low long-term survival rate, and only allogeneic hematopoietic stem cell transplantation (allo-HSCT) can improve the long-term survival of these patients (Zhang et al., 2014; Ren et al., 2019; Hu et al., 2020). However, in clinical practice, post-transplant relapse is still the main factor affecting the long-term survival of patients, and the survival rate after relapse decreases significantly (Yoshimoto et al., 2021). At present, there is no targeted treatment for post-transplant relapse for this group of patients, but common post-transplant relapse treatment strategies are still being adopted, such as donor lymphocyte infusion (DLI), traditional chemotherapy, targeted drug therapy and secondary transplantation. In recent years, immunotherapy for acute lymphoblastic leukemia has made rapid progress and achieved remarkable efficacy, but it is still difficult to obtain satisfactory treatment efficacy in AML patients. Although secondary transplantation has a better response rate, it also has more complications and its long-term survival benefit is insignificant.

Multiple studies have confirmed that, for these patients, the time when the AML1-ETO status becomes positive again post transplantation is the best time for pre-emptive treatment, which can improve the long-term survival of patients (Elmaagacli et al., 1997; Wang et al., 2014; Qin et al., 2017). To improve patient tolerance of treatment, possible adverse reactions must be reduced. Our center uses a 3-drug combination regimen based on chidamide (chidamide, decitabine and interferon (IFN)-α2b) for AML1-ETO patients with early post-transplant relapse. In this study, we sought to explore the safety and efficacy of the combined regimen for AML1-ETO patients with molecular relapse post transplantation.

## 2 Methods

### 2.1 Population

In this single-center, single-arm, non-randomized retrospective study, a total of 24 patients with AML1-ETO–positive AML were enrolled, including 10 men and 14 women; of these, three patients had a consistently positive AML1-ETO status before transplantation. All patients received a modified conditioning regimen containing Flu/BU/CY/ATG-F and underwent bone marrow and peripheral blood or peripheral HSCT at our center between January 2018 and January 2022, followed by a routine graft-versus-host disease (GVHD) prophylaxis regimen (cyclosporine (CSA) and mycophenolate mofetil (MMF)). The median follow-up time was 25 (6–60) months. The basic information about the enrolled patients is shown in Table 1.

**TABLE 1:** Patients’clinical characteristics.

Characteristic	Number	Percent
Gender		
Female	14	58.3
Male	10	41.7
Type of disease		
AML	20	83.3
Secondary AML	2	8.3
MDS transfer to AML	2	8.3
Molecular genetic changes		
KIT	6	25.0
FLT3-ITD	3	12.5
TET2	2	8.33
RUNX1	2	8.33
ASXL1	2	8.33
Without changes	10	41.7
Status before transplantation		
CR1	20	83.3
CR2	4	16.7
MRD-positive	3	12.5
Type of transplantation		
Sibling-matched	6	25.0
Haploidentical	16	66.7
Unrelated	2	8.33
Achieved MCR or not after treatment?		
Yes	19	79.2
No	5	20.8

### 2.2 Transplantation

See our previous article for details (Xi et al., 2021).

### 2.3 Regimen

All patients received induction therapy with a 3-drug combination regimen based on chidamide, as follows: 1) 10 mg/d of oral chidamide once daily (Monday to Saturday); 2) 10 mg of subcutaneously injected decitabine twice weekly (Monday and Tuesday); and 3) 20 μg of subcutaneously administered IFN-α2b every other day (Tuesday, Thursday, and Saturday). The treatment cycle was 4 weeks, and the treatment plans contained at least three cycles. The detection of measurable residual disease (MRD) including molecular and flow cytometry results was performed after treatment. Subjects whose MRD result was complete remission (CR) or partial remission (PR) entered the consolidation treatment period and continued to complete two treatment cycles for consolidation; subjects who experienced disease progression could choose whether to continue to complete the study.

### 2.4 Clinical definition

Efficacy evaluation was conducted for each treatment cycle, including bone marrow cell morphology, bone marrow cell MRD (flow cytometry) result, and bone marrow cell molecular MRD (quantitative polymerase chain reaction, qPCR) result (including AML1-ETO gene and other molecular genetic abnormalities). All experiments were performed by Kangsheng Global Medical Laboratory in accordance with international standard testing procedures.

The efficacy evaluation criteria were as follows: 1) the MRD result turned negative within three treatment cycles, indicating complete remission (MCR); 2) after three treatment cycles, the MRD result improved compared to before treatment but did not turn negative, indicating partial remission (MPR); 3) there was no change in MRD between before treatment and after three cycles of treatment, suggesting the disease was stable (MSD); and 4) the MRD result worsened or even progressed to hematological relapse after the completion of three treatment cycles, indicating disease progression (MPD), which required withdrawal from this clinical study.

### 2.5 Ethics approval

This protocol was re-registered in the Chinese Clinical Research Registry (registration no. ChiCTR2000032330). This study was approved by the Ethics Committee of Sichuan Provincial People’s Hospital, Affiliated Hospital of UESTC (ethics batch no. 2015 Kelun (Vaissiere et al., 2008)). All patients or their guardians signed written informed consent forms to participate in the study in accordance with the Declaration of Helsinki.

### 2.5 Detection of immune cells by flow cytometry

This study focused on changes in immune indexes in patients between before and after treatment. Protocol immunophenotypic analysis was performed for all relapsed patients at baseline time points of 0, 1, 2, and 3 months after the start of the protocol and 3 months after discontinuation. CTL cells were defined as CD3^+^CD4^−^CD8^+^ cells, natural killer (NK) cells were defined aas CD3^−^CD56^+^ cells, T helper (Th) cells were defined as CD3^+^CD4^+^CD8^−^ cells, B-cells were defined as CD3^−^CD19^+^ cells, Th1 cells were defined as CD3^+^CD4^+^CD8^−^INFγ^+^ cells, Th2 cells were defined as CD3^+^CD4^+^CD8^−^IL4^+^, Th17 cells were defined as CD3^+^CD4^+^CD8^−^IL17^+^ cells, and Treg cells were defined as CD3^+^CD4^+^CD25^+^Foxp3^+^ cells.

### 2.6 Data analysis and statistics

GraphPad Prism version 5.0 (GraphPad Software, San Diego, CA, United States) was used for survival analysis and mapping. A *t*-test was used for statistical analysis of measurement data between the two groups, and the chi-squared test was used for statistical analysis of classification data. The cumulative incidence of relapse (CIR), leukemia-free survival (LFS), and overall survival (OS) were measured from the time of relapse post-transplantation by the Kaplan-Meier method and Greenwood’s formula. The log-rank test was used for group comparison. SPSS 13.0 (IBM Corporation, Armonk, NY, United States) was used for these data. *p* < 0.05 was considered to be statistically significant.

## 3 Results

### 3.1 Basic information of patients

A total of 24 patients were enrolled from January 2018 to January 2022. There were 24 AML1-ETO–positive AML patients, including 10 men and 14 women, and three of these patients were in a persistent positive AML1-ETO state before transplantation.

Only AML1-ETO gene were detected in 10 patients, and AML1-ETO accompanied by other molecular genetic abnormalities were detected in 14 patients. The median age of patients in this cohort was 29 years (range, 6–59 years). See Table 1 for details.

### 3.2 Clinical efficacy

**FIGURE 1:**
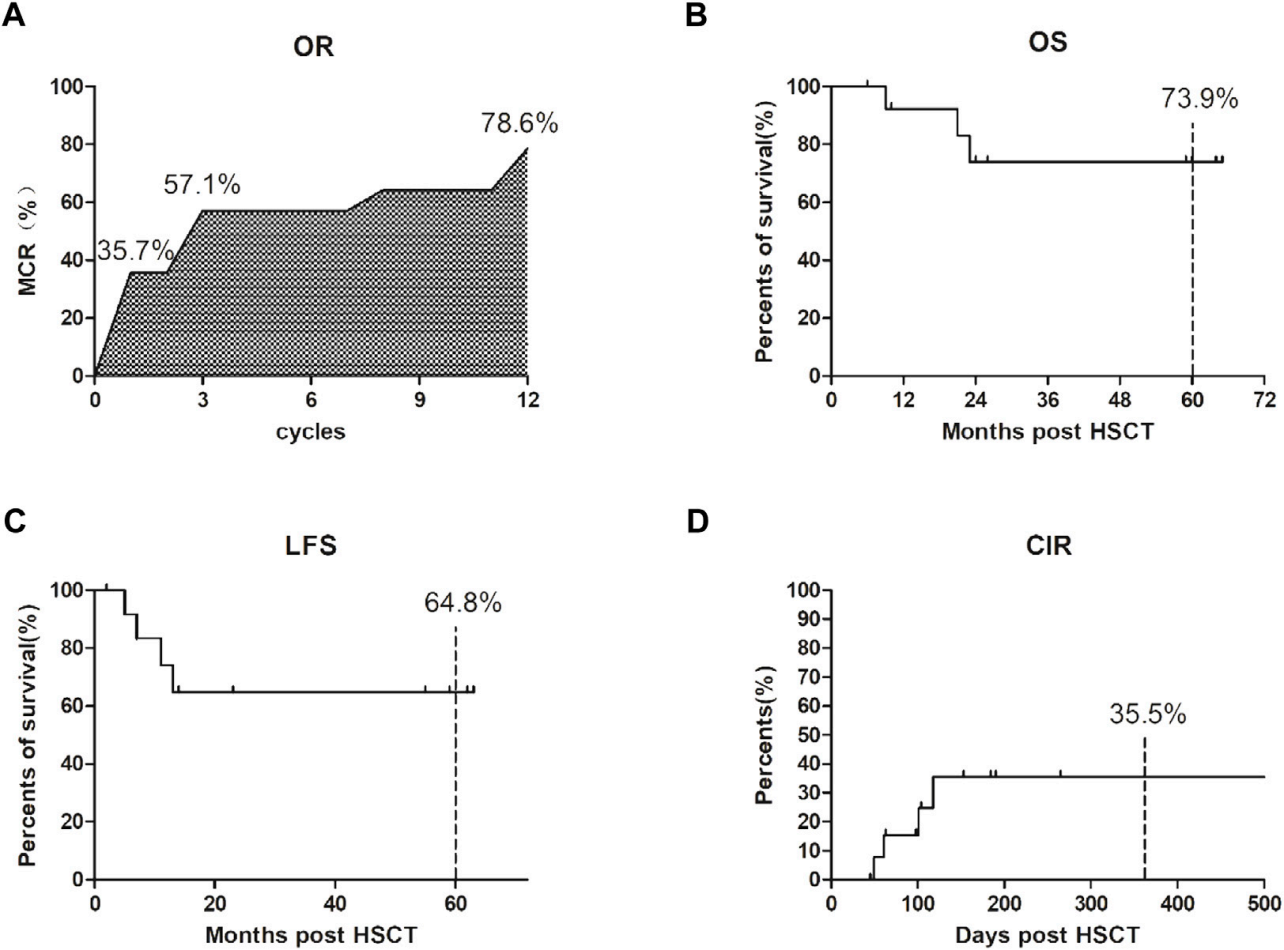
Clinical efficacy after our 3-drug combination regimen based on chidamide. **(A)** The overall response rate (OR) was 95.8% (23/24); The total molecular complete response (MCR) was 79.1% (19/24); the proportion of MCR gradually increases with the increase in treatment courses. **(B)** The projected 5-year overall survival (OS) rate was 73.9%; **(C)** The estimated 5-year leukemia-free survival (LFS) rate was 64.8%; **(D)** The estimated 1-year cumulative relapse rate (CIR) was 35.5%.

In terms of efficacy, AML1-ETO decreased or turned negative in 23 of 24 patients after treatment. The overall response rate was 95.8% (23/24), and the median number of treatment courses was 4 (range, 3–12 courses). The total molecular complete response (MCR) was 79.1% (19/24); more specifically, it was 37.5% (9/24) after one course of treatment but reached 58.3% (14/24) after four courses, suggesting that the proportion of MCR gradually increases with the increase in treatment courses (see Figure 1A and Table 2). The projected 5-year overall survival rate was 73.9% (see Figure 1B). Patients with hematologic relapse died within 6 months, but those with molecular relapse could be managed with continuous treatment. The estimated 5-year leukemia-free survival rate was 64.8% (see Figure 1C), and the estimated 1-year cumulative relapse rate was 35.5% (see Figure 1D).

**TABLE 2:** Dynamics of AM Ll-ETO transcripts post therapy in AML with molecular relapse post transplantation.

Months post this regimen	Number of patients evaluated (%)	Median AML1-ETO transcri • levels (range)	Patients with negative AML1-ET0 (%)
1	24 (100)	0.42% (0%–5.81%)	9 (37.5)
2	24 (100)	0.11% (0%–2.8%)	9 (37.5)
3	24 (100)	0.031% (0%–3.1%)	9 (37.5)
4	24 (100)	0% (0%–8.1%)	14 (58.3)
5	24 (100)	0% (0–3.1%)	14 (58.3)
6	24 (100)	0% (0%–11.2%)	14 (58.3)
7	22 (92.6)	0% (0%–39.3%)	14 (63.6)
8	22 (92.6)	0% (0%–1.1%)	15 (68.2)
9	22 (92.6)	0% (0%–0.25%)	15 (68.2)
10	22 (92.6)	0% (0%–4.3%)	15 (68.2)
11	22 (92.6)	0% (0%–8.9%)	15 (68.2)
12	20 (83.3)	0% (0%–65.3%)	19 (95.0)
18	20 (83.3)	0% (0%–54.6%)	19 (95.0)
24	19 (83.3)	0% (0.0%)	19 (100)
30	19 (83.3)	0% (0%–0%)	19 (100)
36	19 (83.3)	0% (0%–0%)	19 (100)

### 3.3 Adverse reactions

**FIGURE 2:**
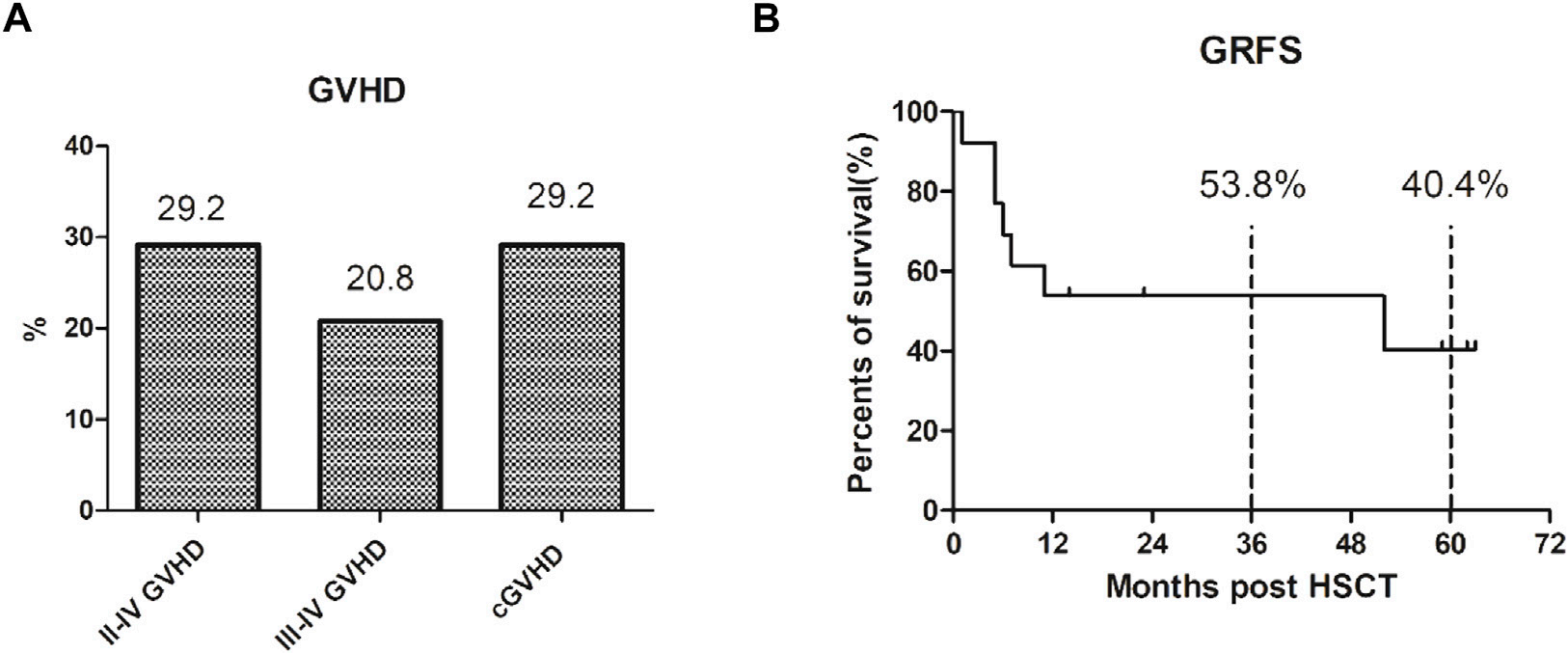
The GVHD and GRFS after our 3-drug combination regimen based on chidamide. **(A)** The overall incidence of III–IV GVHD was 29.2% (7/24), III–IV GVHD was 20.8% (5/24), and the total incidence of chronic GVHD was 29.2% (7/24), which were localized chronic GVHD. **(B)** The projected 3-year GRFS is 53.8% and 5-year GRFS is 40.4%.

Among the 24 patients, seven developed varying degrees of GVHD (see Figure 2A), mainly presenting as skin rejection and liver rejection. The overall incidence of GVHD was 29.2% (7/24), among which the incidence of grade III–IV GVHD was 20.8% (5/24), and cases could be effectively controlled by glucocorticoid therapy combined with calcination inhibitors. The total incidence of chronic GVHD was 29.2% (7/24), and all cases were localized chronic GVHD. The projected 5-year GRFS is 40.4% (see Figure 2B).

Eight patients developed different degrees of pulmonary infection, with an infection rate of 33.3% (8/24). The main pathogens were bacteria and fungi. One patient developed a serious life-threatening pneumocystis *pneumoniae* infection (4.17%, 1/24), which improved after active treatment and respiratory support. There were no treatment-related deaths in the 24 patients, and the treatment-related mortality rate was 0%. The overall mortality rate was 20.8% (5/24). Nausea and vomiting, elevated aminotransferase levels, thrombocytopenia, and neutropenia are common adverse reactions, all of which were Common Terminology Criteria for Adverse Events grade 2–3 and could be reversed after drug withdrawal or combined hepatoprotective therapy.

### 3.4 Changes in immune constituents

**FIGURE 3:**
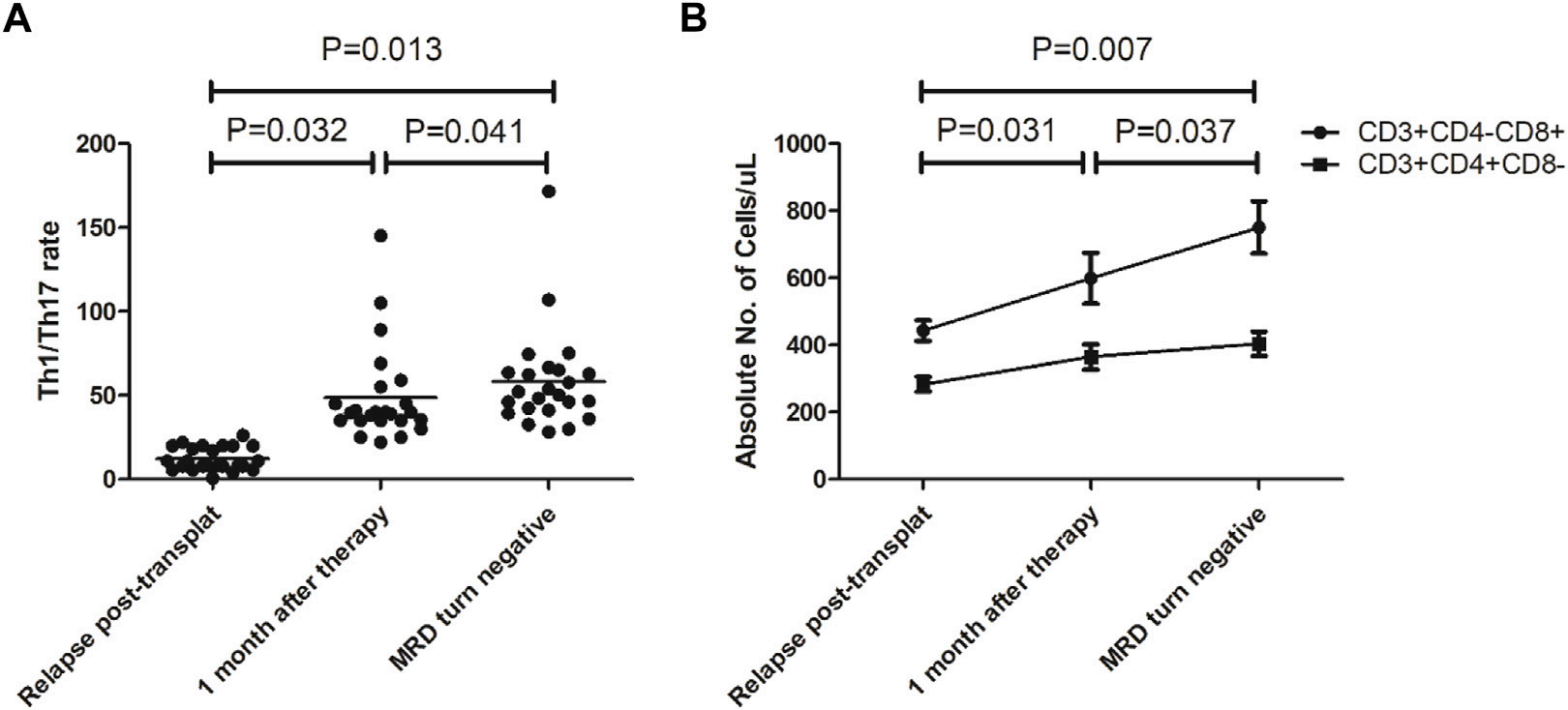
Changes in immune constituents after our 3-drug combination regimen based on chidamide. **(A)** Th1/Th17 ratio gradually increased compared to that before treatment; **(B)** The CD8^+^ T lymphocyte count increased gradually, while the CD4^+^ T lymphocyte count did not change significantly.

The cellular immune constituents of patients were dynamically monitored before and after treatment. The results showed Th1 cell counts gradually increased, Th17 cell counts gradually decreased, and the Th1/Th17 ratio gradually increased compared to that before treatment (see Figure 3A). The CD8^+^ T lymphocyte count increased gradually, while the CD4^+^ T lymphocyte count did not change significantly (see Figure 3B).

## 4 Discussion

Currently, the commonly used treatment options for post-transplant relapse include secondary allo-HSCT (Choi et al., 2021; Yalniz et al., 2021), donor lymphocyte infusion (Zhao et al., 2022), traditional chemotherapy or targeted drug therapy (Yoshimoto et al., 2021), and chimeric antigen receptor T-cell (CAR-T) and cellular immunotherapy (Cui et al., 2021). There is still no safe and effective treatment for post-transplant relapse in AML1-ETO patients, and secondary transplantation has been reported to have the highest remission rate. Choi et al. (2021) reported that 80 patients with acute leukemia who had relapsed post allo-HSCT (HSCT1) were treated again with allo-HSCT (HSCT2). Patients with genetic testing results included 54 AML patients, including 44 patients at low or moderate risk (the number of patients with AML1-ETO was not given). The results showed that the median overall survival and event-free survival were 10.3 and 6.1 months after HSCT2, respectively, and acute GVHD cases numbered as high as 28 (35.0%), while grade 3–4 acute GVHD cases totaled as high as 11 (13.8%). While chronic GVHD was found in 27 patients, generalized chronic GVHD was found in 17 patients. Moreover, severe liver veno-occlusive disease (VOD) was found in seven patients (8.8%). At the same time, studies have shown that patients who relapsed less than 6 months after HSCT1 or who failed to achieve a CR/CR with incomplete CR (CRi) result after HSCT2 could not benefit from HSCT2. Acute and chronic GVHD cases associated with HSCT2 increase significantly, especially for patients with relapse of HSCT1 among 6 months. This data suggests it is difficult to benefit from HSCT2, whereas HSCT2 will greatly increase the economic burden of patients. Zhao et al. (2022) enrolled 26 patients with relapsed AML after allo-HSCT, including 18 patients in the medium-risk group, who were treated with venetoclax, azacytidine, and DLI. CR/CRi and PR rates were 26.9% and 34.6% post treatment, respectively. The median event-free survival and overall survival were 120 days and 284.5 days, respectively; however, six patients (23.1%) developed severe GVHD. With the progress of medical science, new treatments are also emerging, such as CD38-targeted CAR-T therapy (Cui et al., 2021). Although this new immunotherapy strategy achieved a high response rate early in treatment, its cumulative relapse rate at 6 months was up to 50%, and its median overall survival and leukemia-free survival times were 7.9 and 6.4 months, with a higher risk of cytokine release synthesis (CRS). In addition, this study was a small sample study with selection bias in enrolled patients, so it is still worth exploring whether this approach can be used as the optimal treatment strategy for patients with post-transplant relapse. In conclusion, for AML1-ETO positive AML patients post transplantion, the existing treatments have poor efficacy and many adverse reactions, including especially III-IV GVHD. It is extremely urgent to explore a safe and effective regimen for these post-transplant relapse patients.

Epigenetic modifications, such as DNA methylation and histone and non-histone acetylation, are important regulators of all aspects of T-cell life and are involved in T-cell development, differentiation, and activation. In recent years, studies have found that epigenetic regulation disorders are closely related to the pathogenesis of AML, in that > 70% of AML patients have DNA methylation–related gene mutations or histone modification mutations, and demethylation drugs can effectively reverse such abnormal changes (Mehdipour et al., 2015). Reduced histone acetylation mediated by histone deacetylase (HDAC) is another common epigenetic anomaly, and targeted inhibition of HDAC reverses dysacetylation. In patients with AML1-ETO, ETO replaces the c-terminal regulatory regions of activation and inhibition of wild-type AML1 in its encoded protein. ETO binds to co-inhibitory factors and recruits HDAC1, 2, and three to form co-inhibitory complexes, thus silencing target genes and preventing hematopoietic differentiation and transformation. It also interacts with secondary mutations, including C-KIT, FLT3, and RAS, ultimately leading to the occurrence or progression of AML1-ETO–positive leukemia (Zhang et al., 2021). The chidamide-based 3-drug combination regimen adopted in this study is based on the theory of correcting epigenetic disorders. As a commonly used HDAC inhibitor (HDACi), chidamide can selectively inhibit HDAC1, 2, 3, and 10, and it is widely used in T-cell lymphoma, advanced breast cancer, and other diseases. Decitabine is a commonly used clinical demethylation drug, which is widely used in myelodysplastic syndrome, acute myeloid leukemia, and refractory immune thrombocytopenia. Basic studies have shown that HDACis interact with DNA methylation, and HDACis help remove methyl-CPG–binding proteins (MePC2) from methylated cytosine. HDACi therapy also allows histone acetyltransferases to re-acetylate histones at gene promoters, and highly acetylated histones can recruit DNA demethylases to further protect DNA from methylation (Vaissiere et al., 2008). For AML1-ETO, HDACi degrades this fusion protein by upregulating E2 ubiquitin binding enzyme and E3 ubiquitin ligase (Buchwald et al., 2009). In *in vitro* studies, chidamide inhibited the proliferation of AML1-ETO–positive AML cells, induced cell cycle arrest, and stimulated apoptosis. At the same time, histone three acetylation and ERK1/2 phosphorylation of AML1-ETO–positive AML cells were inhibited, and the ERK1/2 pathway was regulated to inhibit the proliferation of AML1-ETO–positive AML cells and c-kit expression was reduced (Liu et al., 2020). Of note, immune escape is another mechanism of poor outcome in patients with AML1-ETO–positive leukemia. Elias et al. (2014) found the AML1-ETO gene downregulated the expression of CD48 on the surfaces of leukocytes, thereby reducing the killing effect of natural killer cells, while HDACi can promote the re-expression of CD48 and restore the sensitivity of leukemia cells to natural killer cells. Demethylation drugs also have a similar effect on promoting immune cell recognition (Ganetsky, 2012). Therefore, double-epigenetic treatment has a synergistic effect; on the one hand, it directly exerts anti-leukemia effects, while on the other, it promotes the immune system to better recognize tumor cells and enhance the ability to eliminate leukemia cells. This anti-leukemia mechanism is especially suitable for patients post-HSCT, which gives full play to amplify the graft *versus* leukemia (GVL) effct.

IFN-α, as a type I IFN, is approved by the United States. Food and Drug Administration for the treatment of various malignant blood tumors and solid tumors. In this study, the addition of IFN-α to the epigenetic therapy strategy is an important improvement of the previous epigenetic regulation therapy. Over the past few decades, increasing evidence showed that IFN-α can promote type-I anti-tumor effects (Th1-mediated), playing a key role in anti-tumor immune responses. Medrano R. et al. (Medrano et al., 2017) found that 69 of 100 patients with recurrent acute leukemia post allo-HSCT in a single center (AML72, ALL28) received chemotherapy/supportive therapy, with an average survival time of 51 days. Eleven patients were treated with DLI, and the mean survival time was 84 days. Thirteen patients underwent secondary transplantation, with an average survival time of 303 days; seven patients were treated with IFN-α2b combined with GM-CSF, and the mean survival time was 442 days. The efficacy of this group was significantly better than that in other treatment groups. In mechanism studies, several reports have explored the role of IFN-α in the treatment of relapsed AML post transplantation (Mo et al., 2018; Molica et al., 2019; Magenau et al., 2021). First, IFN-α was found to have direct anti-tumor effects on AML cells: it 1) inhibited the secretion of growth promoting factors; 2) stimulated apoptosis, 3) inhibited the proliferation of tumor cells, and 4) increased the immunogenicity of AML cells. Second, IFN-α was found to have indirect anti-tumor effects on AML cells through the 1) activation of natural killer cells and T-cells; 2) by enhancing the antigen-presentation of dendritic cells, and 3) showing a similar effect to that of interleukin-15, which can induce dendritic (DC) cells to become natural killer (NK) cells, thus directly producing cytotoxic effect on AML cells. These experimental results support the multiple roles of IFN-α in anti-AML. Thus, presumably, in our 3-drug combination, the effect of IFN-α was multifaceted, which may synergy with chidamide and decitabine. The mechanism has yet to further define by experiment researches.

## 4 Conclusion

There are limited treatment options for patients with relapse post allo-HSCT. Rapid and sustained reversion of AML1-ETO leukemia relapse post transplantation is the key to improving the long-term survival. Chidamide, as a common HDACi, combining with decitabine and IFN-α2b, is an effective regimen to reverse the molecular biological relapse of AML1-ETO leukemia post transplantation, with a high remission rate and significantly lower adverse reactions than current strategies for post-transplantation relapse. It is worth further extending clinical researches to evaluate the efficacy of this regimen.

## Data Availability

The raw data supporting the conclusions of this article will be made available by the authors, without undue reservation.
